# Increasing the Reproducibility of Science through Close Cooperation and Forking Path Analysis

**DOI:** 10.3389/fpsyg.2017.01332

**Published:** 2017-08-03

**Authors:** Jan Wacker

**Affiliations:** Department of Psychology, Universität Hamburg Hamburg, Germany

**Keywords:** reproducibility, replication, researcher degrees of freedom, scientific practice, false positives, statistical power, cooperative forking path analysis

Within multiple fields alarming reproducibility problems are now obvious to most: The majority of the reported effects are either false positives or the population effect size is much smaller than expected based on the initial studies (e.g., Ioannidis, 2005; Button et al., 2013; Open Science Collaboration, 2015; Baker, 2016; Nichols et al., 2017). Assuming that neither outright scientific fraud (Fanelli, 2009) nor severe deficits in methodological training are the norm, likely reasons for this inacceptable status quo include the following: (A) a high prevalence of severely underpowered studies (e.g., Button et al., 2013), (B) hypothesizing after results are known (HARKing; Kerr, 1998), (C) intentionally or unintentionally exploiting researcher degrees of freedom (Simmons et al., 2011) in data processing and analysis and thereby pushing the *p*-value of statistical tests below the conventional significance level without being transparent concerning all the variables and approaches that have been tried out (P-HACKING), and (D) selective reporting of research findings and publication bias. Several options for pre-registration of hypotheses are now readily available providing the opportunity to effectively prevent HARKing (e.g., OSF.io, AsPredicted.org). However, suggestions to address the other three issues have so far met with the following challenges:

- A practical approach to promote cooperative data collection as the default approach in everyday scientific practice is lacking. Increasing sample sizes enough to ensure appropriate statistical power not only requires more financial resources, it is also more time-consuming. Investing more money per study to increase power is inevitable. However, for work involving restricted access to methodology and/or lengthy paradigms the time requirements may oftentimes make it undesirable or even impossible for individual researchers to conduct appropriately powered studies. Sharing the load of data collection among several labs is an obvious solution, albeit one that is currently mostly limited to large international consortia (e.g., Thompson et al., 2014).
- A practical approach to systematically draw on the joint expertise of larger and more diverse groups of researchers in designing studies is currently lacking. Both *selective reporting and publication bias* can be quite effectively reduced by combining pre-registration of both hypotheses and all relevant details of the research design with a system of peer-review and in-principle acceptance for publication *before* data collection. However, this approach typically lays the burden of deciding for the most appropriate research design on a small number of authors from one work group. In addition, reviewers of research designs by authors within the same field may not always be optimally motivated to detect and correct potential design flaws, because they compete with these authors for grants and faculty positions.
- A practical approach to maintaining flexibility in data analysis, while at the same time ensuring the absence of p-hacking and assessing the influence of various data processing and analysis decisions is currently lacking. The benefits of methodological guidelines, pre-registration of analysis steps, transparency, and open data are indisputable (Nosek et al., 2015). However, limiting researcher degrees of freedom through methodological guidelines and pre-registration of all processing/analysis steps comes at the price of reduced flexibility in adopting novel approaches and in dealing with unexpected data patterns. Especially for studies with large-scale data collections lasting for months or even years the requirement to stick to a pre-registered analysis plan may get into conflict with the desire to make reasonable adjustments according to recent methodological or empirical developments.
  Transparency concerning all variables assessed and all analysis decisions made may aid a highly motivated and/or specialized reader in identifying relevant researcher degrees of freedom (for a list of 34 researcher degrees of freedom see Wicherts et al., 2016). Furthermore, when this transparency principle is combined with open access to all relevant raw data, readers may even have the opportunity to verify hypotheses concerning the relevance of certain processing and analysis decisions for the final outcome. However, investing considerable amounts of time in probing an individual paper's approach to the data is currently neither rewarded during the review process nor does it typically add much to the reputation of researchers. Under these circumstances and given the high rate of publication in most fields of empirical research it is likely that individual empirical papers will rarely receive the level of scrutiny necessary to identify relevant but undisclosed researcher degrees of freedom. Moreover, even a shattering critique of a highly visible paper may not receive sufficient attention to effectively avert other researchers from building their own work on the questionable results.

To address these issues and to increase the reproducibility of scientific findings in multiple fields cooperative forking path analysis (cFPA) studies may be a useful scientific standard for empirical research. cFPA studies may be complemented by open access to data, pre-registration of hypotheses, and in-principle acceptance before data collection and they adhere to the following five principles:

COOPERATIVE. cFPA studies are conducted in teams consisting of researchers from different laboratories that agree on a set of research questions and have access to laboratory equipment allowing them to produce relevant data in a sufficiently similar format. Members of a cFPA team work jointly on all steps of scientific research from specifying the research questions to writing up the final report. Teams are formed informally aided by (online) social networks and aiming to maximize the *diversity* of theoretical and methodological preferences while maintaining a constructive and rewarding work atmosphere. Shared authorship of all team members on grant proposals and papers with first authorship of the person who initially proposed the general idea, initiated the collaborative endeavor, and took the lead writing (both the grant proposal and) the manuscript may often be a useful ground rule.
AGREED-UPON DESIGN. cFPA teams openly and thoroughly discuss (in person or in an online forum) and then agree upon a precise formulation of all research hypotheses and all specific features of the research design relevant to data collection. Whenever an agreement cannot be reached, the team goes through with the majority vote while all minority votes are documented along with the names of the team members supporting them. The final version of the agreed-upon design is summarized as a sufficiently detailed guideline to direct data collection within each of the laboratories. Minority votes are inserted as footnotes in the appropriate sections of the guidelines and the guidelines are published as online supplementary methods.
MULTICENTER. cFPA teams share the load of data collection equally among laboratories with *each* laboratory contributing *at least* enough data to achieve sufficient statistical power (e.g., 1-beta = 0.80) to detect a “large” effect (e.g. *r* = 0.50) with the conventional criterion of alpha = 0.05. Total sample size (and thus the minimum number of contributing laboratories) is determined such that high statistical power (>0.95) is achieved for a conservative estimate of the population effect size of interest, which will usually not exceed the average size of replication effects in a given field (e.g., *r* = 0.20 in Psychology, see Open Science Collaboration, 2015). All data are converted to an equivalent format and then integrated into a single data file including laboratory identifiers and information on all potentially relevant variables that vary across laboratories before all analyses.
SCRIPTED PROCESSING AND ANALYSIS. All processing and analysis steps from the raw data to the tables and figures in the final publication are fully automatized and do not require any user intervention apart from starting the respective routines. Whenever possible this is done with open source software and algorithms to ensure complete transparency at every step (for examples from neuroimaging literature see Waskom et al., 2014; Whitaker et al., 2016).
FORKING PATHS ANALYSIS. cFPA teams openly and thoroughly discuss every single step in data processing and analysis aiming to identify as many defensible alternative approaches as possible while taking votes on which approach should be used in the present study. If necessary, this may be facilitated by collectively working with a representative subsample of data randomly drawn from each of the contributors and together amounting to the sample size collected by a single lab; in that case, data from the subsample used to establish analysis procedures is not included in the final analysis, but should either be made openly available or analyzed separately in a supplement. Each defensible processing and analysis approach is scripted (preferably in open source code). This process continues until all forking analysis paths arrive at their final statistical test of the effect of interest. (Note that even with relatively complex fMRI methods the number of defensible alternative statistical tests for a given effect of interest does not seem to exceed 35,000; Carp, 2012). The one path consisting of the processing and analysis steps that the majority of the cFPA team agreed upon (and that includes appropriate tests of and controls for inter-laboratory variance) is then flagged as the result to be written up for publication. However, along with this result the relative frequency of defensible processing and analysis paths leading to the same significant finding is reported. Also, the most influential processing and analysis decisions are labeled, for instance, with a beta weight indicating the degree to which the final effect size changed due to the respective analysis decision and with the level of agreement among the team members. The complete table of effect size beta weights and non-anonymous vote counts could be published as a supplement.

cFPA studies bear at least the following advantages:

*Reduced likelihood of fraud and questionable research practices*. The level of social control inherent in having several laboratories cooperate for data collection and analysis helps to reduce the likelihood of fraud and questionable research practices. Also, the conception of science as a fundamentally cooperative process may run a lower risk of attracting individuals motivated more by narcissistic motives (e.g., outshining others with a stream of baffling ideas and findings) rather than gaining knowledge.
*Increased statistical power*. cFPA studies aim for sample sizes at least 10 times the sample size of a study with sufficient statistical power only for large effects within the same time frame. The resulting reduction in the percentage of severely underpowered studies would lead to a decrease in the likelihood of false positive findings and to less exaggerated effect size estimates (Loken and Gelman, 2017). Also, because larger samples cost more, fewer but larger studies would be conducted if the cFPA principles were widely adopted, which could ultimately lessen the burden on (grant) reviewers and readers trying to keep track of the relevant literature in their field.
*Facilitated identification of best practices*. Publications of cFPA studies routinely include a systematic assessment of inter-laboratory variance and (if this variance component is significant) of the influence of specific differences across labs (e.g., laboratory equipment) that may explain part of this variance. The consensual data processing and analysis approach as well as the most influential decisions concerning single steps in the stream are documented in a highly accessible fashion. These features could aid considerably in identifying best practices of data acquisition, processing and analysis, and they are formative for research in the field.
*Facilitated direct replications*. The requirement to devise highly specific data collection guidelines that are later published as supplementary material along with the research findings will facilitate direct replications.
*Facilitated assessment of reproducibility*. Final processing and analysis paths containing one or even more nodes with extremely high beta weights for standardized changes in effects sizes due to methodological choices (especially ones that were controversial within the group) may aid in identifying reproducibility issues.
*Facilitated and more focused communication on relevant methodological issues*. The requirement to conduct expert discussions among individuals with diverse views for both planning the study and processing/analyzing the data will foster communication on important methodological issues, helping to disseminate superior methodological innovations and standards more quickly (especially if these discussions were integrated as new formats in scientific conferences).

For these reasons it seems that multiple areas of empirical science could benefit considerably from doing more cFPA studies. The principles described here may be instrumental in reducing waste of research efforts in a wide range of contexts ranging from low-cost collaborative student projects to large collaborative research projects with substantial funding.

## Author contributions

The author confirms being the sole contributor of this work and approved it for publication.

### Conflict of interest statement

The author declares that the research was conducted in the absence of any commercial or financial relationships that could be construed as a potential conflict of interest. The reviewer SL and handling Editor declared their shared affiliation, and the handling Editor states that the process nevertheless met the standards of a fair and objective review
